# Impact of discharge medication counseling in the cardiology unit of a tertiary hospital in Brazil: A randomized controlled trial

**DOI:** 10.6061/clinics/2018/e325

**Published:** 2018-04-17

**Authors:** Aline F. Bonetti, Bruna Q. Bagatim, Antonio M. Mendes, Inajara Rotta, Renata C. Reis, Maria Luiza D. Fávero, Fernando Fernandez-Llimós, Roberto Pontarolo

**Affiliations:** I Departamento de Farmacia, Universidade Federal do Parana, Curitiba, PR, Brasil; II Departamento de Socio-Farmacia, Instituto de Pesquisa de Medicamentos (iMed. Ulisboa), Faculdade de Farmacia, Universidade de Lisboa, Lisboa, Portugal

**Keywords:** Patient Discharge, Medication Adherence, Patient Readmission, Counseling, Pharmacists

## Abstract

**OBJECTIVES:**

This study aimed to evaluate the impact of pharmacist-provided discharge counseling on mortality rate, hospital readmissions, emergency department visits, and medication adherence at 30 days post discharge.

**METHODS:**

This randomized controlled trial was approved by the local ethics committee and included patients aged 18 years or older admitted to the cardiology ward of a Brazilian tertiary hospital. The intervention group received a pharmacist-led medication counseling session at discharge and a telephone follow-up three and 15 days after discharge. The outcomes included the number of deaths, hospital readmissions, emergency department visits, and medication adherence. All outcomes were evaluated during a pharmacist-led ambulatory consultation performed 30 days after discharge.

**RESULTS:**

Of 133 patients, 104 were included in the analysis (51 and 53 in the intervention and control groups, respectively). The intervention group had a lower overall readmission rate, number of emergency department visits, and mortality rate, but the differences were not statistically significant (*p*>0.05). However, the intervention group had a significantly lower readmission rate related to heart disease (0% *vs*. 11.3%, *p*=0.027), despite the small sample size. Furthermore, medication counseling contributed significantly to improved medication adherence according to three different tools (*p*<0.05).

**CONCLUSIONS:**

Pharmacist-provided discharge medication counseling resulted in better medication adherence scores and a lower incidence of cardiovascular-associated hospital readmissions, thus representing a useful service for cardiology patients.

## INTRODUCTION

Medication errors are an important risk factor for hospitalization and mortality 1,2. Several factors contribute to increased readmission rates, including time to primary care follow-up, adverse drug events 3,4, medication nonadherence 5, medication discrepancies 6,7, insufficient discharge planning 8, and lack of continuity in care 9. Medical conditions associated with high rates of re-hospitalization include congestive heart failure, acute myocardial infarction (AMI), pneumonia, diabetes, and chronic obstructive pulmonary disease 9.

Up to 67% of patients admitted to hospitals have medication discrepancies that often persist at discharge 10. One observational study reported that patients with medication discrepancies had a 30-day hospital readmission rate of 14.3%, compared with 6.1% in patients without discrepancies 6. The consequences of these issues affect patient safety as well as the quality and cost of care 6,11. The World Health Organization estimates that only 50% of individuals with chronic diseases adhere to their medication regimen 12. Recent studies have demonstrated that adherence to cardioprotective medications, such as statins and clopidogrel, is poor in the first year after acute coronary syndrome. After AMI, nearly 24% of patients do not fill their medications within seven days of discharge, and one-third of patients decide to stop at least one medication in the first month 5,13.

Pharmacists may play a significant role in the transition of care, especially in reducing readmissions by ensuring that appropriate, evidence-based pharmacotherapy regimens have been prescribed during hospitalization, identifying medication errors, and performing medication reconciliation 8.

The results of several studies suggest that medication counseling before hospital discharge reduces the incidence of patient medication discrepancies and adverse drug events 4,7. Counseling also improves adherence 14,15, decreases readmission rates 1,16 and mortality 5, and consequently results in cost savings 17,18; however, other reports have suggested that counseling has little or no effect 19,20.

Medication counseling can ensure that patients are appropriately educated about their medications. However, most previous studies have been conducted in high-income countries, and the endpoints were evaluated via telephone interviews. Therefore, the aim of this trial was to assess the impact of pharmacist-led medication counseling at hospital discharge on mortality rate, hospital readmissions (related and unrelated to heart disease), emergency department visits (related and unrelated to heart disease), and medication adherence in a developing country.

## METHODS

This randomized controlled trial was performed according to CONSORT guidelines and was conducted in a tertiary hospital in Curitiba, Brazil, from February to December 2015. This hospital has 360 beds and admits approximately 30 patients per month on the cardiology ward. Patients aged 18 years and older were eligible if they were admitted to a specialized cardiology ward due to stable angina, acute coronary syndrome, congestive heart failure, valvular disease, arrhythmias, or hypertension. The exclusion criteria included patients with cognitive impairment and without a caregiver, palliative care status, and subjects who refused to participate in this study. Patients transferred to other clinical specialties or institutions and those who were not discharged from the hospital before the end of the study were also excluded.

The sample size was calculated considering 5.0% type I and 20% type II error (80% power) rates based on hospital readmission rates reported in the literature, resulting in 53 patients in each group. Following the randomization procedures, the participants were randomly assigned to one of two groups. Eligible patients who provided informed consent were allocated to either the intervention group or control group in a 1:1 ratio using a random number list generated by a third person using *Microsoft Office Excel 2010*^®^. The sequence of inclusion corresponded to the hospital admission order. Two cardiovascular pharmacy residents were responsible for patient enrollment according to the eligibility criteria and for performing the intervention. These pharmacy residents were in the department daily, and they received coaching during the first year of residency.

Patients who were allocated to the intervention group or their caregivers received individual counseling sessions regarding the discharge prescriptions. These sessions included a thorough assessment of the pharmacotherapy and interventions from cardiologists in order to correct any medication issues, as well as an explanation about the indications, benefits, therapeutic targets, dose, dosing schedule, routes, storage, length of therapy, refill pharmacy, and possible adverse drug events for each prescribed drug. A leaflet containing the information provided in the verbal counseling was delivered by the pharmacists. Subsequently, patients were contacted by telephone three and 15 days post-discharge to reinforce the previous counseling session. All pharmacist interventions were performed and described according the Descriptive Elements of Pharmacist Interventions Characterization Tool (DEPICT) 21.

The control group received usual care from pharmacists and other healthcare providers. During the hospitalization, all patients, including those from the control group, received pharmaceutical interventions as necessary. At the time of hospital discharge, patients from both groups were scheduled for an appointment 30 days post-discharge in the Pharmaceutical Care Ambulatory Clinic of the same hospital. During this appointment, the pharmacist team for the ambulatory clinic collected data regarding the participant outcomes and performed counseling for both groups as necessary. Subsequently, all patients were followed equally by the ambulatory clinic. There were five trained pharmacists in this setting, including one of the residents who provided the intervention.

The primary endpoints were mortality rate, hospital readmissions (related and unrelated to heart disease), and emergency department visits (related and unrelated to heart disease) within 30 days. We asked the patients whether they visited emergency departments during this period and whether they were admitted to another hospital.

The secondary endpoint was medication adherence based on the results of the *MedTake* 22, Beliefs about Medicines Questionnaire (BMQ) 23, and Adherence to Refills and Medications Scale (ARMS) 24 instruments, all completed 30 days post-discharge. The *MedTake* test is a quantitative assessment of drug-taking procedures for oral prescriptions that is used to screen for medication adherence problems. The test evaluates dosage (number of dosage units), indications, food or water co-ingestion, and regimens. Each test is scored as the percentage of correct actions (0%: zero adherence; 100%: total adherence) 22. To better interpret the overall results, we divided the results into *MedTake* 1, and *MedTake* 2, defined as the percentage of drugs that the patient was taking properly and the percentage of drugs for which the patient was aware of the indications, respectively. The BMQ is a questionnaire composed of two subscales: a five-item necessity scale to assess beliefs about the necessity for medication and a six-item concerns scale to assess beliefs about the danger of dependence and the disruptive effects of medication. Each item is scored on a five-point scale (1=strongly disagree, 2=disagree, 3=uncertain, 4=agree and 5=strongly agree); the final result is obtained by the quotient between the percentage of the sum of “necessity” and “concern” questions 23. Higher scores indicate greater awareness of the necessity for taking the medications, decreased concern regarding the possible negative effects, and better medication adherence. Finally, the ARMS instrument is a medication adherence scale intended for patients with chronic medical conditions. It includes a total of 14 items, each of which is structured with responses “none,” “some,” “most,” or “all” of the time, which are assigned values from 1 to 4, respectively. The questionnaire contains questions about the frequency of medication withdrawal in healthcare institutions (“refill”) and about forgetfulness in taking medications (“taking”) 24. Patients with better medication adherence have scores near 12, whereas patients with no adherence have scores of 48.

Statistical analyses were conducted using SPSS Statistics for Windows, version 22.0. Kolmogorov-Smirnov tests were used to assess the normality of the distribution of the investigated parameters. Student’s *t* and Mann-Whitney tests were used for continuous data as appropriate, and categorical variables were assessed using chi-square and Fisher’s exact tests. *P* values lower than 0.05 were considered statistically significant.

### Ethics approval

This trial was in performed accordance with the standards of the institution’s ethics committee (approval number: 40431015.8.0000.0096), the national research committee, and the 1983 Helsinki declaration and its later amendments or comparable ethical standards.

## RESULTS

**Figure 1:**
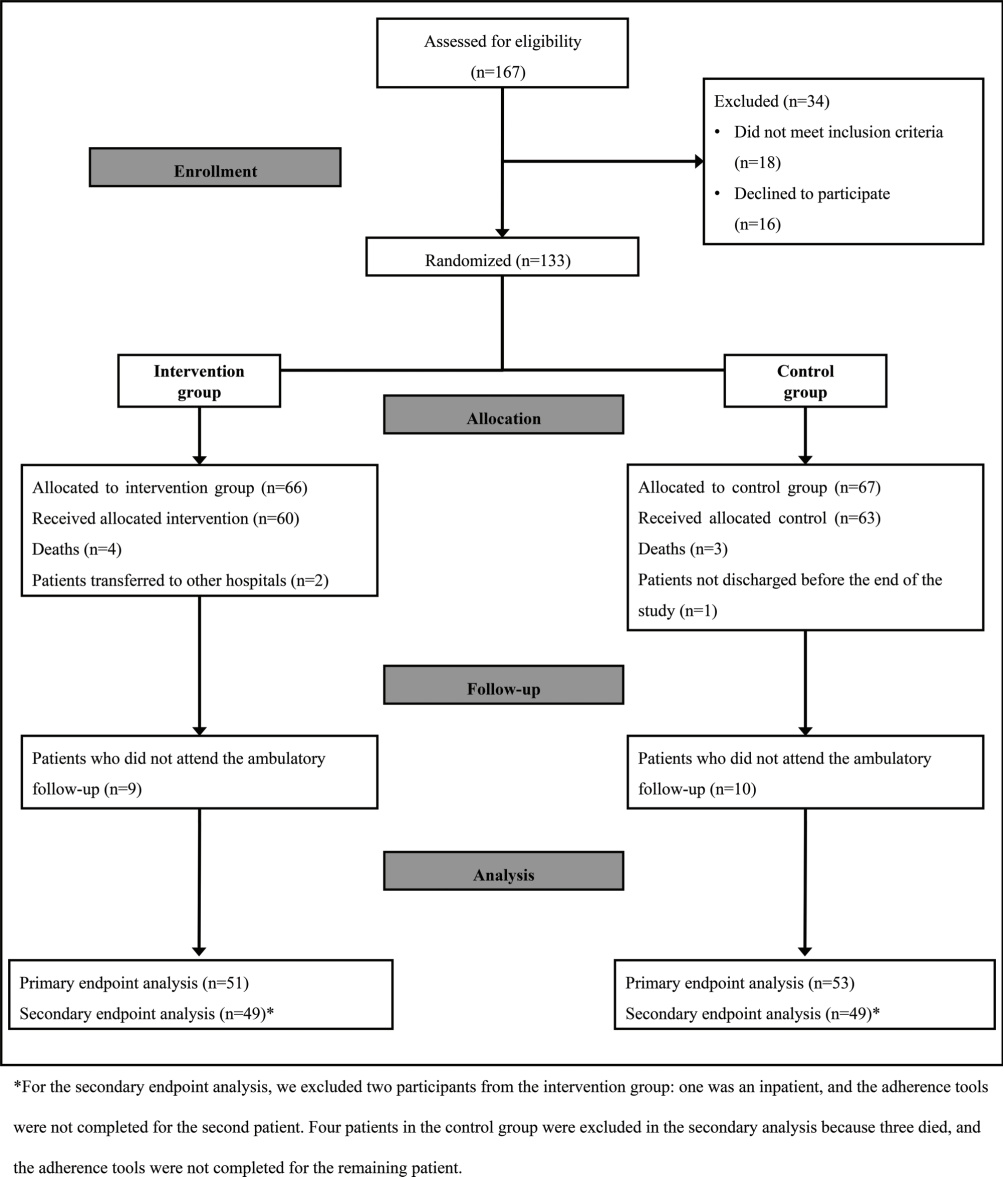
Flow diagram of the intervention and control groups in the randomized trial.

Of the 167 eligible patients recruited between February and November 2015, 133 agreed to participate, and 34 were excluded. A total of 66 and 67 patients were allocated to the intervention and control groups, respectively, and patient follow-up occurred through December 2015 (Figure 1).

There were no statistically significant differences in baseline characteristics between the two groups (Table 1). The mean age of the participants was 65 years, most were men, and all of the participants were on polypharmacy. The most frequent reason for hospitalization was AMI, followed by unstable angina, congestive heart failure, and atrial fibrillation. The most common comorbidities were hypertension, diabetes, and dyslipidemia.

There were three deaths during the 30 days post-discharge, all in the control group. These patients were included in the primary endpoint analysis, but the difference between the intervention and control groups was not statistically significant (*p*=0.243; Table 2).

The rate of hospital readmissions related to heart disease was significantly lower in the intervention group than in the control group (*p*=0.027). Overall readmissions and emergency department visits related to heart disease were higher in the control group, but the differences were not statistically significant (*p*=0.374 and *p*=0.118, respectively; Table 2).

The mean scores of each adherence instrument revealed that the intervention group was significantly more adherent to medications than the control group (*p*<0.05; Table 3).

## DISCUSSION

This study revealed that medication counseling at discharge decreased early readmission rates, especially related to heart disease, and improved medication adherence. These outcomes were evaluated during a pharmaceutical appointment, unlike previous studies, indicating the potential for pharmacotherapeutic follow-up of patients. This is the first study to evaluate the impact of medication counseling on readmission rates, emergency department visits, deaths, and medication adherence using three different tools.

According to similar studies, pharmacist-led medication review, patient counseling, and telephone follow-up are associated with lower readmission rates after hospital discharge; however, some of these results were not statistically significant 19,25-27, and some of these studies were conducted as non-randomized trials 1,15,27.

A prospective study reported that older age, history of diabetes, a greater number of prescriptions before AMI, and history of congestive heart failure are associated with increased one-year mortality. Patients who received discharge counseling from pharmacists had a lower risk of one-year mortality (*p*=0.001) 5. Our study did not observe a statistically significant reduction in mortality among patients with these characteristics; however, we evaluated early mortality in a randomized controlled trial design, whereas the previous study was a cohort design that analyzed one-year mortality following AMI.

A similar randomized trial revealed that patients who received discharge medication counseling from nurses and telephone follow-up from pharmacists exhibited lower rates of hospital utilization than usual care participants (*p*=0.009), but there was no difference in early readmission rates (*p*=0.090) 17. Another study evaluated the impact of the pharmacy team in transition-of-care settings and demonstrated reduced composite emergency department visits and inpatient readmissions (*p*=0.022) but reported no significant differences in hospital readmission rates separately (*p*=0.43) 28. Conversely, our study revealed lower 30-day readmission rates due to cardiac diseases in patients who were allocated to the intervention group, emphasizing that medication counseling may be important for cardiology patients with polypharmacy to reduce readmission rates caused by improper use of medication.

We also observed that discharge counseling and pharmacotherapy follow-up by phone were essential for medication adherence. The intervention group had significantly higher mean total *MedTake*, *MedTake* 1, and *MedTake* 2 percentages compared with the control group, indicating that the pharmacist-led counseling contributed to an increased patient understanding of the medication-taking process and the indications for each medication. Other factors may contribute to medication adherence, including patient resources, attitudes, beliefs, perceptions, and expectations towards medication 29. However, we observed that the intervention group was significantly more adherent to their medications than the control group based on the BMQ and ARMS results.

The limitations of this study include a small patient population that, despite exceeding the minimum sample size calculations, may have resulted in an underestimation of the effect of the intervention. However, our trial is underpowered to adequately study mortality, other causes of hospital readmissions, and emergency department visits. We note that not everyone in the ambulatory setting was blinded to the study allocation, but the primary outcomes are objective and consequently were not affected by the lack of blinding. Another limitation is that the intervention group could have been affected by the Hawthorn effect, contributing to the positive results.

The results of this study revealed that discharge medication counseling performed by pharmacists decreased 30-day hospital readmissions, particularly those related to cardiac disease, and improved medication adherence in cardiology patients under polypharmacy. Transition of care is a high-risk situation for many patients, and abundant discharge counseling is needed. The involvement of pharmacists in the transition of care is advisable to reduce re-hospitalization related to inappropriate use of medications.

## AUTHOR CONTRIBUTIONS

Bonetti AF and Pontarolo R had full access to all of the study data and take responsibility for the integrity of the data and the accuracy of the data analysis. Bonetti AF, Bagatim BQ, Mendes AE, Reis RC, and Favero ML were responsible for the study concept and design. Bonetti AF, Bagatim BQ and Mendes AE were responsible for the acquisition of the data. Bonetti AF, Mendes AE and Rotta I were responsible for the analysis and interpretation of the data. Bonetti AF, Bagatim BQ and Mendes AE were responsible for the manuscript drafting. Bonetti AF, Bagatim BQ, Mendes AE, Rotta I, Reis RC, Favero ML, Fernandez-Llimos F and Pontarolo R were responsible for the critical revision of the manuscript for important intellectual content. Fernandez-Llimos and Pontarolo were responsible for the study supervision.

## Figures and Tables

**Table 1:** Patient characteristics at baseline.

Variable*	Intervention (n=51)	Control (n=53)	p***
Age (years)	65 (±10)	65 (±13)	0.939
Men, n (%)	35 (68.6)	34 (64.2)	0.629
Number of comorbidities	4 (±1)	4 (±2)	0.929
Number of medications at discharge	7 (±2)	8 (±3)	0.430
Number of medications at ambulatory follow-up**	8 (±3)	9 (±3)	0.250
Days of hospitalization	10 (±9)	12 (±9)	0.273
Presence of caregiver, n (%)	14 (28)	17 (34)	0.517

* Mean and SD are reported for continuous data.

** n=49 in each group.

*** Statistically significant if *p*<0.05.

**Table 2:** Primary endpoint results.

Endpoints*	Intervention (n=51)	Control (n=53)	p**
Emergency department visits related to heart disease	0	4 (7.5)	0.118
Emergency department visits not related to heart disease	3 (5.9)	1 (1.9)	0.289
Total hospital readmissions	4 (7.8)	7 (13.2)	0.374
Hospital readmissions related to heart disease	0 (0)	6 (11.3)	0.027
Hospital readmissions not related to heart disease	4 (7.8)	1 (1.9)	0.156
Deaths	0 (0)	3 (5.7)	0.243

* Data are reported as absolute and relative numbers (%).

** Statistically significant if *p*<0.05.

**Table 3:** Secondary endpoint results.

Endpoints*	Intervention (n=49)	Control (n=49)	p**
Total *MedTake*	92.1 (±9.9)	58.5 (±31.9)	<0.001
*MedTake* 1	95.2 (±8.6)	64.7 (±37.1)	<0.001
*MedTake* 2	85.2 (±21.5)	44.5 (±34.8)	<0.001
BMQ	1.8 (±0.6)	1.6 (±0.5)	0.028
ARMS	13 (±2)	15 (±4)	0.001

* Mean and SD are reported for the data.

** Statistically significant if *p*<0.05.

Abbreviations: ARMS: Adherence to Refills and Medications Scale; BMQ: Beliefs about Medicines Questionnaire.
